# Integrated machine learning and multi-omics analysis identifies CYP1B1 as a candidate target of orientin in intervertebral disc degeneration-associated low back pain

**DOI:** 10.3389/fpain.2026.1870398

**Published:** 2026-06-24

**Authors:** Hai-E Liu, Dong-Dong Guo, Kun Liu, Xing-Jing Luo

**Affiliations:** Department of Anesthesiology, National Children’s Medical Center, Children’s Hospital, Fudan University, Shanghai, China

**Keywords:** low back pain, molecular docking, network pharmacology, orientin, ScRNA-seq analysis

## Abstract

**Objectives:**

Orientin is a bioactive agent isolated from natural resources successfully applied for pain control. This study was to explore candidate hub gene of orientin to intervertebral disc degeneration (IDD) -associated low back pain.

**Methods:**

This study integrated the network pharmacology analysis using orientin and IDD-associated pain, machine learning and immune infiltration analysis using GSE70362 as prediction analysis. The validation was performed via molecular docking, molecular dynamics simulation, single-cell RNA sequencing (scRNA-seq) analysis and cell experiment using human annulus fibrosus cells under IL-1β stimulation.

**Results:**

Network pharmacology analysis showed that there were 368 overlapped genes between orientin and IDD-associated low back pain. Machine learning demonstrated that Cytochrome P450 1B1 (CYP1B1) was candidate hub genes with excellent diagnostic performance (AUC = 0.95). Immune infiltration analysis showed that CYP1B1 had no correlation with infiltration levels of any immune cell subset cells after Benjamini-Hochberg false discovery rate correction. Molecular Docking showed docking energies of orientin to CYP1B1 were all smaller than −9.5 kcal/mol. The molecular dynamics simulation showed a system of orientin-CYP1B1 showed a relatively stable conformational ensemble. scRNA-seq analysis revealed tissue- and cell-type specific expression of CYP1B1, with significant upregulation in annulus fibrosus fibroblasts from diseased samples. In cell experiments, orientin alleviated the decrease in cell viability induced by IL-1β stimulation, and down-regulated CYP1B1 gene expression and CYP1B1 protein levels.

**Conclusion:**

This study showed that CYP1B1 is a potential candidate hub gene for orientin to IDD-associated low back pain.

## 1 Introduction

Low back pain is an age-dependent disease with a prevalence rate of 4.39% worldwide, causing pain, movement limitation and disability in some severe subjects (1). The lifetime prevalence rate is more than 80% (1), which means the low back pain has a long duration and causes long-term pain for patients, resulting in great social and economy burden. Low back pain is a typical age-associated chronic pathological condition. The gradual degeneration of intervertebral discs (2), inflammation (3), anti-oxidation stress unbalance (4) were some contributors but pathological factors were not limited to biopsychosocial and lifestyle factors. The incidence rates in women were higher than men (5), while women after menopause are higher compared with others (6). Up to now, the pathological mechanism of low back pain remain incompletely clarified due to its complexity (7).

Orientin is a water-soluble ﬂavonoid C-glycoside. In the past decade, it was found as a natural agent with pharmacology bioactivities (8), anti-inflammation (9), anti-oxidative (10). It has a variety of injury and organ protection effects, neuroprotective, antiaging, cardioprotective, antidiabetic and hepatoprotective (11). Studies have found that natural plants or extracts containing orientin often have analgesic activity (12). Glucosylated flavones that contain orientin and others exert anti-inflammatory and analgesic activities from *Vitex polygama* (13). Importantly, our team first demonstrated its analgesic effect on neuropathic pain in animal models (14). Subsequently, we further validated its analgesic effect on neonatal pain animal model through network pharmacology prediction, molecular docking and animal experiment validation (15). Two years afterward, another oversea independent team also confirmed that it can effectively inhibit neuropathic pain (16). Therefore, orientin maybe a candidate drug with multiple analgesic effects to several pain conditions. Most importantly, another study revealed that orientin inhibited the endoplasmic reticulum stress and mitochondrial dysfunction via AMPK/SIRT1 pathway in cell experiment and ameliorated IDD in animal experiment (17). The previously study hinted the possible analgesic application to IDD-associated low back pain conditions because the IDD, inflammation and oxidative stress are the contributors of IDD-associated low back pain (17). This study employed a machine learning–based integrated model combined with multiple bioinformatics analyses to predict core targets associated with IDD–related low back pain, and further evaluated their binding potential through molecular docking and molecular dynamics simulations.

## 2 Materials and method

**Figure 1:**
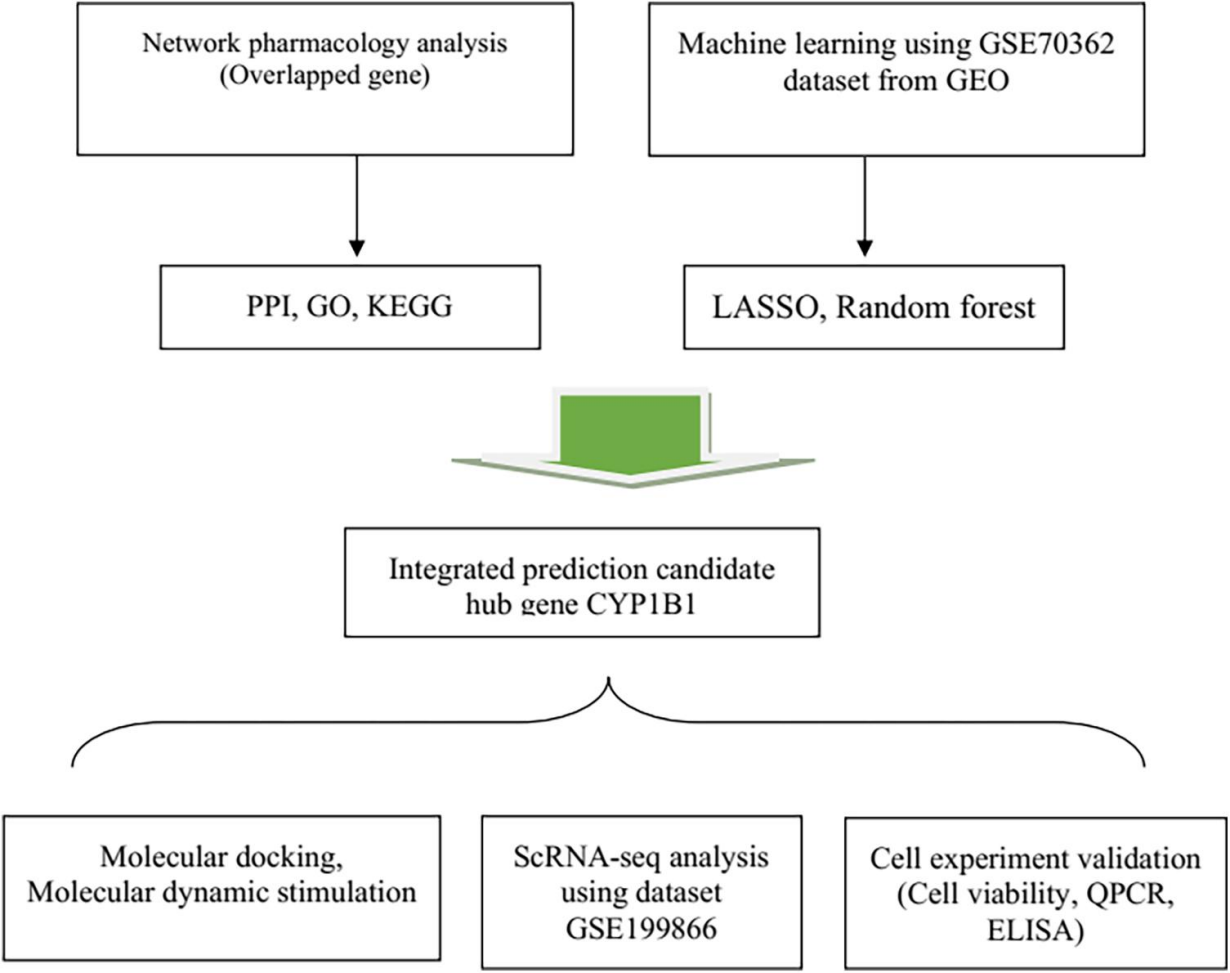
Flowchart of the overall study design.

The flow chart and process were detailed in Figure 1, which contains an integrated prediction of network pharmacology analysis, machine learning, immune infiltration, a validation of molecular docking, molecular dynamics simulation and cell experiments.

### 2.1 Network pharmacology analysis

#### 2.1.1 Preparation of orientin and low back pain targets

The purpose of this step was to comprehensively identify potential therapeutic targets of orientin and disease-associated genes for subsequent network construction. Orientin related targets were obtained using a standardized multi-source integration strategy adapted from our previously published work (15), with an updated search conducted on September 12, 2025. The potential targets of orientin (PubChem CID: 5281675) were retrieved from some widely used pharmacology databases: Traditional Chinese Medicine Systems Pharmacology Database (TCMSP, https://www.tcmsp-e.com/#/database) (18), Swiss Target Prediction Database (https://swisstargetprediction.ch/) (19), and Super-PRED database (https://prediction.charite.de/index.php?site=chemdoodle_search_target) (20). For Swiss Target Prediction, the Simplified Molecular-Input Line-Entry System (SMILES) string of orientin (O1[C@]([H])(C([H])([H])O[H])[C@]([H])([C@@]([H])([C@]([H])([C@]1([H])C1 = C(C([H])=C(C2C(C([H])=C(C3C([H])=C([H])C(=C(C = 3[H])O[H])O[H])OC1 = 2)=O)O[H])O[H])O[H])O[H])O[H]) was input, the probability threshold was set to > 0.5 (the database's default high-confidence cutoff), and the species was limited to Homo sapiens. All retrieved targets were converted to official gene symbols using the UniProt Knowledgebase (21) (UniProtKB, Release 2025_04, https://www.uniprot.org/), with only manually reviewed Swiss-Prot entries retained and duplicate targets removed by matching UniProt IDs. Next, disease targets associated with low back pain were downloaded from 2 authoritative disease databases on September 15, 2025: GeneCards (22) (Version 5.16, https://www.genecards.org/), Online Mendelian Inheritance in Man (OMIM) (23) (Updated September 10, 2025, https://www.genecards.org/). For GeneCards, the search term “low back pain” was used, the species was limited to Homo sapiens, and only targets with a relevance score ≥ 5.0 (the database's default threshold for high-relevance disease genes) were included. For OMIM, the search term “low back pain” was used with the “All Fields” option, and only genes with confirmed disease associations marked as “confirmed” in the database were retained. All disease targets were similarly converted to official gene symbols using UniProtKB. The common therapeutic targets of orientin against low back pain were identified by intersecting the two gene lists using Venn diagram web tool (Venny 2.1 https://bioinfogp.cnb.csic.es/tools/venny/index.html), and all common targets were further verified for consistency in gene nomenclature and UniProt ID mapping before subsequent analysis.

#### 2.1.2 Network construction and analysis

The Protein-protein interaction (PPI) network (24) of the overlapped genes was to identify protein-level interactions among overlapping targets and to prioritize hub genes based on topological importance, constructed and drawn via the STRING database (25) (Version 12.0, accessed September 12, 2025, https://www.string-db.org/) to access potential core target networks, with the species limited to Homo sapiens, the minimum required interaction score set to 0.9 (the highest confidence level), all default interaction sources enabled including text mining, experiments, databases, co-expression, neighborhood, gene fusion, and co-occurrence, disconnected nodes removed, and self-interactions excluded. Visualization of the PPI network was performed using Cytoscape 3.8.2 (26) and cytoHubba plugin (27) (Version 0.1).

#### 2.1.3 Gene ontology and Kyoto encyclopedia of genes and genomes enrichment analysis

Gene Ontology (GO) and Kyoto Encyclopedia of Genes and Genomes (KEGG) pathway prediction was to elucidate the biological processes, cellular components, molecular functions, and signaling pathways potentially involved in orientin's action against low back pain*.* The overlapped targets between orientin and low back pain were subjected to Metascape database (28) (Version 3.5, accessed September 12, 2025, https://metascape.org/gp/index.html#/main/step1) for GO and KEGG pathway prediction using the setting of gene “OFFICIAL_GENE_SYMBOL” and species “homo sapiens”, with the background gene set to the whole human genome (database default), statistical test being performed via hypergeometric test, and *P*-value correction being conducted by Benjamini-Hochberg false discovery rate (FDR) method. This database automatically performed the GO analysis in terms of molecular function (MF), biological processes (BP), and cellular components (CC). The statistical graph was generated using default setting (Min overlap threshold at 3, *P* Value Cutoff 0.01, Min Enrichment threshold at 1.5).

### 2.2 Machine learning

To screen and prioritize the most robust hub genes from the overlapping targets, machine learning was initiated at proper dataset selection using the reported criteria: at least 40 samples, accessible raw data and others (29). The microarray GEO dataset GSE70362 (30) which contains transcriptome data from 48 intervertebral disc tissue samples (12 controls and 36 IDD-associated low back pain subjects) used in this study, was employed to explore the expression levels of IDD-associated low back pain related targets.

The machine learning analysis employed 2 machine learning algorithms including Least Absolute Shrinkage and Selection Operator (LASSO) regression using glmnet packages and random forest algorithms to identify core target genes following previously report (31). GSE70362 dataset was randomly split into a training set (70%, *n* = 35) and an independent test set (30%, *n* = 13) using stratified sampling to preserve the group distribution. For each candidate gene, the optimal cutoff value was determined using the Youden index on the training set, with weights of 0.4 for sensitivity and 0.6 for specificity to prioritize diagnostic specificity. The diagnostic performance was evaluated on the held-out test set using the area under the receiver operating characteristic curve (AUC), sensitivity, specificity, positive predictive value (PPV), and negative predictive value (NPV). The 95% confidence intervals (CIs) for AUC were calculated using the percentile method with 10,000 stratified bootstrap resamples to ensure each resample contained both disease and control samples. The statistical significance of the AUC was assessed using a permutation test with 10,000 permutations, which is particularly robust for small sample sizes. All statistical analyses were performed in R version 4.3.3.

### 2.3 Immune infiltration assay

Immune infiltration analysis was performed using the CIBERSORT algorithm with the standardized LM22 human immune cell signature matrix to estimate the relative infiltration fractions of 22 immune cell subtypes in degenerative intervertebral disc tissues, thereby characterizing the immune cell composition and exploring potential correlations between hub gene expression and immune cell infiltration. Considering the inherently low baseline immune cell abundance within intervertebral disc parenchyma, CIBERSORT was prioritized over enrichment-based tools (including GSVA, ssGSEA and xCell), as its linear support vector regression-based deconvolution approach enables more stable quantification of subtle immune transcriptional signals in immune-poor solid tissues. After generating infiltration fraction matrix from raw gene expression data, Wilcoxon rank-sum test was applied to statistically compare infiltration differences of each immune cell subtype between low-back-pain and control groups, with visualization of differential infiltration conducted via boxplots and stacked bar charts. Spearman correlation analysis was further performed to explore the relationships between candidate hub genes and the infiltration levels of distinct immune cell subtypes, and correlation heatmaps were constructed to present the overall association landscape.

### 2.4 Molecular docking validation

The purpose of molecular docking was to computationally predict the binding affinity and interaction mode between orientin and the candidate hub gene CYP1B1.The AutoDock Vina (version 1.2.2) was used for molecular docking to evaluate the binding energy and interaction mode between orientin and Cytochrome P450 1B1 (CYP1B1). The x-ray structure 3PM0 of CYP1B1 was obtained from the PDB database. The SDF format 3D structure of orientin (CAS: 28608-75-5) was obtained from the PubChem database. The protein and ligand files were prepared separately. For protein preparation, water molecules and heteroatoms except the native ligand were removed from the 3PM0 structure, polar hydrogen atoms were added, and Kollman charges were assigned using AutoDock Tools (ADT, version 1.5.7). The protein was saved in PDBQT format. For ligand preparation, the 3D structure of orientin was energy-minimized using the Merck Molecular Force Field 94 (MMFF94) force field, then processed in ADT to assign Gasteiger charges, detect rotatable bonds, and save in PDBQT format. The same protocol was applied to the native ligand *α*-naphthoflavone (extracted from 3PM0) for redocking validation. A docking grid box of 28 Å × 28 Å × 28 Å was centered on the geometric center of the co-crystallized *α*-naphthoflavone (center coordinates: x = –21.138, y = 20.917, z = –19.865). AutoDock Vina 1.2.2 was used with an exhaustiveness value of 64, num_modes = 10, and energy_range = 4. The root-mean-square deviation (RMSD) between the redocked pose and the crystal structure was 0.00 Å, confirming the reliability of the protocol. Subsequently, orientin was docked into the same binding site using the validated protocol, and the best binding pose was selected based on the lowest binding energy. PLIP and LigPlot + were used to systematically analyze the binding interface of the protein–ligand complex, and PyMOL 2.5 software (32) was used to supplement the interaction-related details.

### 2.5 Molecular dynamics simulation validation

Molecular dynamics simulations of orientin-CYP1B1 complex and apo-CYP1B1 were performed using GROMACS 2022.5 software to validate the binding stability and structural effects of orientin on the core target CYP1B1 of low back pain. The crystal structure of human CYP1B1 (PDB ID: 3PM0) was prepared by adding hydrogen atoms, assigning partial charges, and repairing missing residues. The three-dimensional structure of orientin was converted to a GROMACS-compatible format using Open Babel, and its topological parameters were defined using a CHARMM36-compatible ligand force field to ensure consistency with the protein force field. The protein was parameterized with the CHARMM36 force field, which is suitable for high-precision biomolecular simulations.

The system was solvated in a cubic box with a minimum distance of 1.0 nm between the protein surface and the box boundary, and filled with TIP3P water molecules. Counterions (Na⁺/Cl⁻) were added to neutralize the system charge and achieve a physiological ionic strength of 0.15 M. Energy minimization was performed in two steps: first, the Steepest Descent algorithm was applied to relax the system and remove high-energy instabilities, reducing the maximum force below 1000 kJ/mol/nm; second, the Conjugate Gradient algorithm was used for further refinement to resolve residual van der Waals and electrostatic clashes and converge to a stable low-energy conformation.

Subsequently, two equilibration simulations were conducted. A 100 ps isothermal isovolumetric (NVT) simulation was performed at 300 K using the V-rescale thermostat, with position restraints applied to the protein backbone atoms to allow full thermal equilibration of the solvent and system. This was followed by a 100 ps isothermal isobaric (NPT) simulation at 300 K and 1 bar using the Parrinello-Rahman barostat, with backbone restraints gradually released to stabilize the system volume.

Three independent 100 ns production simulations were performed for orientin-CYP1B1 complex with different initial velocities, and one 100 ns simulation was performed for the apo-CYP1B1 protein under identical conditions as a control. The time step was set to 2 fs. Long-range electrostatic interactions were calculated using the Particle Mesh Ewald (PME) method, and the short-range van der Waals interactions were truncated at 1.2 nm. All chemical bond lengths were constrained using the LINCS algorithm to allow stable integration of hydrogen proton motion. Trajectory coordinates were saved every 10 ps, and state parameters including energy, temperature, and pressure were recorded every 1 ps for subsequent analysis.

Convergence of the simulations was rigorously validated using both instantaneous and cumulative average backbone root-mean-square deviation (RMSD) analyses. The cumulative average RMSD was calculated to provide a robust assessment of thermodynamic equilibrium. All quantitative analyses were performed using only the well-equilibrated segment from 60 ns to 100 ns, ensuring a unified thermodynamic equilibrium interval for comparison between the complex and apo systems. The following parameters were analyzed: (1) backbone RMSD relative to the initial structure to assess overall protein stability, (2) radius of gyration (Rg) to evaluate protein structural compactness, (3) intermolecular hydrogen bonds between orientin and CYP1B1 (for the complex) and intramolecular hydrogen bonds (for the apo system), defined by a donor-acceptor distance ≤ 0.35 nm and a bond angle ≥ 120°. The Molecular Mechanics Poisson-Boltzmann Surface Area (MM/PBSA) method was used to calculate the binding free energy of orientin to CYP1B1, using 400 evenly spaced snapshots extracted from the equilibrated trajectories, and per-residue decomposition was performed to identify key contributing residues. Statistical analyses were conducted using Python 3.10. Data are presented as mean ± standard deviation (SD). Independent samples t-tests (Welch's *t*-test) were used to compare differences in RMSD, Rg, and hydrogen bond number between the complex and apo systems, with *P* < 0.05 considered statistically significant.

### 2.6 Single cell RNA sequencing experiment

The purpose of scRNA-seq analysis was to examine CYP1B1 expression at single-cell resolution across different cell types in NP and AF tissues from IDD associated low back pain patients and controls. The GSE199866 public dataset from the 10X Genomics platform was used for scRNA-seq. It contains nucleus pulposus (NP) samples from degenerating discs with IDD-associated low back pain (Disease group) and sample of disc non degenerating without low back pain (Control group), also a pair of annulus fibrosis sample in disease group and sample of control group. All single-cell RNA-seq data were analyzed using R (v4.3.3) with the Seurat package. Shared genes across samples were retained, and unique barcodes were generated to avoid duplication before merging count matrices. For quality control, cells with fewer than 100 detected genes and genes expressed in fewer than 3 cells were filtered out. The dataset was normalized using the LogNormalize method, and 2500 highly variable genes were selected via the variance-stabilizing transformation algorithm, followed by global scaling and centering. Principal component analysis (PCA) was performed, and the top 20 principal components were used for subsequent analysis. Uniform Manifold Approximation and Projection (UMAP) was applied for dimensionality reduction and visualization. Cell clustering was conducted using the Louvain algorithm with a resolution of 0.6. Cell types were annotated by calculating module scores of canonical marker gene sets, and each cell was assigned to the cell type with the highest score. Four core visualizations were generated: combined UMAP plots showing cell distribution by group and unsupervised clusters, UMAP plot of annotated cell types, stacked bar plot presenting the relative proportion of each cell type between groups, as well as feature plots and violin plots comparing the expression levels of target genes CYP1B1. All graphs were visualized using ggplot2 and pheatmap packages.

To further evaluate the statistical significance of CYP1B1 expression differences between disease and control groups across distinct cell populations, Wilcoxon rank-sum test was performed, with false discovery rate (FDR) correction applied for multiple testing.

### 2.7 Cell experiment

#### 2.7.1 Cell culture, model establishment, and orientin treatment

Human annulus fibrosus cells (HAFCs, Cat.: CSC-I9316L, Species: Homo sapiens, Source: Disc Tissue, Morphology: Fibroblast-like, Creative Bioarray, USA) were cultured in specialized annulus fibrosus cell medium supplemented with 10% fetal bovine serum and 1% penicillin-streptomycin at 37°C in a humidified incubator with 5% CO₂. To establish an *in vitro* model, HAFCs were stimulated with 10 ng/mL recombinant human IL-1β (PeproTech, USA) for 24 h. Cells were randomly divided into five groups. The Control group was cultured without any treatment. The Model group was treated only with 10 ng/mL IL-1β for 24 h. For orientin intervention groups, cells were co-incubated with 10 ng/mL IL-1β and orientin at three different concentrations for 24 h. Orientin (purity ≥ 98%, Sigma-Aldrich, USA) was used at final concentrations of 15.6 μM, 31.2 μM and 62.4 μM, which were defined as low-, medium- and high-dose groups, respectively. Except for the Control group, IL-1β and orientin were added simultaneously to the culture medium and incubated for 24 h. The Control group was supplemented with an equivalent volume of vehicle (0.1% DMSO, final concentration ≤ 0.01%).

#### 2.7.2 Cell viability

Concisely, HAFCs cells were harvested after 24 h exposure. The cell viability was calculated by previously method (33). The absorbance value of each well was measured at the specified wavelength using a microplate reader, and the relative cell viability was calculated by comparing the optical density of the treatment groups with that of the untreated control group. This assay quantitatively reflects cellular metabolic activity and was used to screen safe and effective drug concentrations for subsequent functional experiments.

#### 2.7.3 QPCR of CYP1B1 mRNA levels

Briefly, primers of CYP1B1 were prepared and the process of QPCR was according to previous reports (34). Quantitative real-time PCR was performed to detect the mRNA expression level of CYP1B1 in HAFCs. Total cellular RNA was extracted from treated cells after 24 h of intervention, and RNA purity and concentration were determined before reverse transcription into complementary DNA (cDNA). Specific primers targeting CYP1B1 were designed and synthesized, and the quantitative real-time polymerase chain reaction (qRT-PCR) amplification procedure was performed in accordance with a previously established protocol (35). Relative gene expression levels were normalized to the internal reference gene and calculated.

#### 2.7.4 The enzyme-linked immunosorbent assay (ELISA) of CYP1B1 levels

After 24 h exposure, cell culture was collected and centrifuged to remove cell debris. The concentrations of CYP1B1 were detected strictly following the manufacturer's instructions and a previously reported experimental procedure (34). The absorbance of each sample was measured using a microplate reader, and the cytokine concentrations were calculated based on standard curve.

### 2.8 Statistical analysis

All bioinformatics analyses were performed in R version 4.3.3. The principal R packages used were: Seurat (v4.4.0) for single-cell data processing, limma (v3.58.1) for differential expression analysis, glmnet (v4.18) for LASSO regression, randomForest (v4.71.1) for random forest analysis, pROC (v1.18.5) for ROC curve analysis, ggplot2 (v3.5.1) and pheatmap (v1.0.12) for data visualization. All cell experiments were conducted in three independent biological replicates, and the results were presented as mean ± 95% CI. Statistical analyses were performed using SPSS software (Version 27.0). One-way analysis of variance (one-way ANOVA) was used for overall comparison among groups. The homogeneity of variance was tested prior to *post-hoc* analysis. The least significant difference (LSD) test was applied for multiple comparisons when variance homogeneity was satisfied. If the variance was heterogeneous, Dunnett's T3 test was adopted instead. *P* value less than 0.05 was considered to indicate a statistically significant difference.

## 3 Results

### 3.1 Network pharmacology prediction

#### 3.1.1 Screening of target genes associated with orientin and low back pain

**Figure 2:**
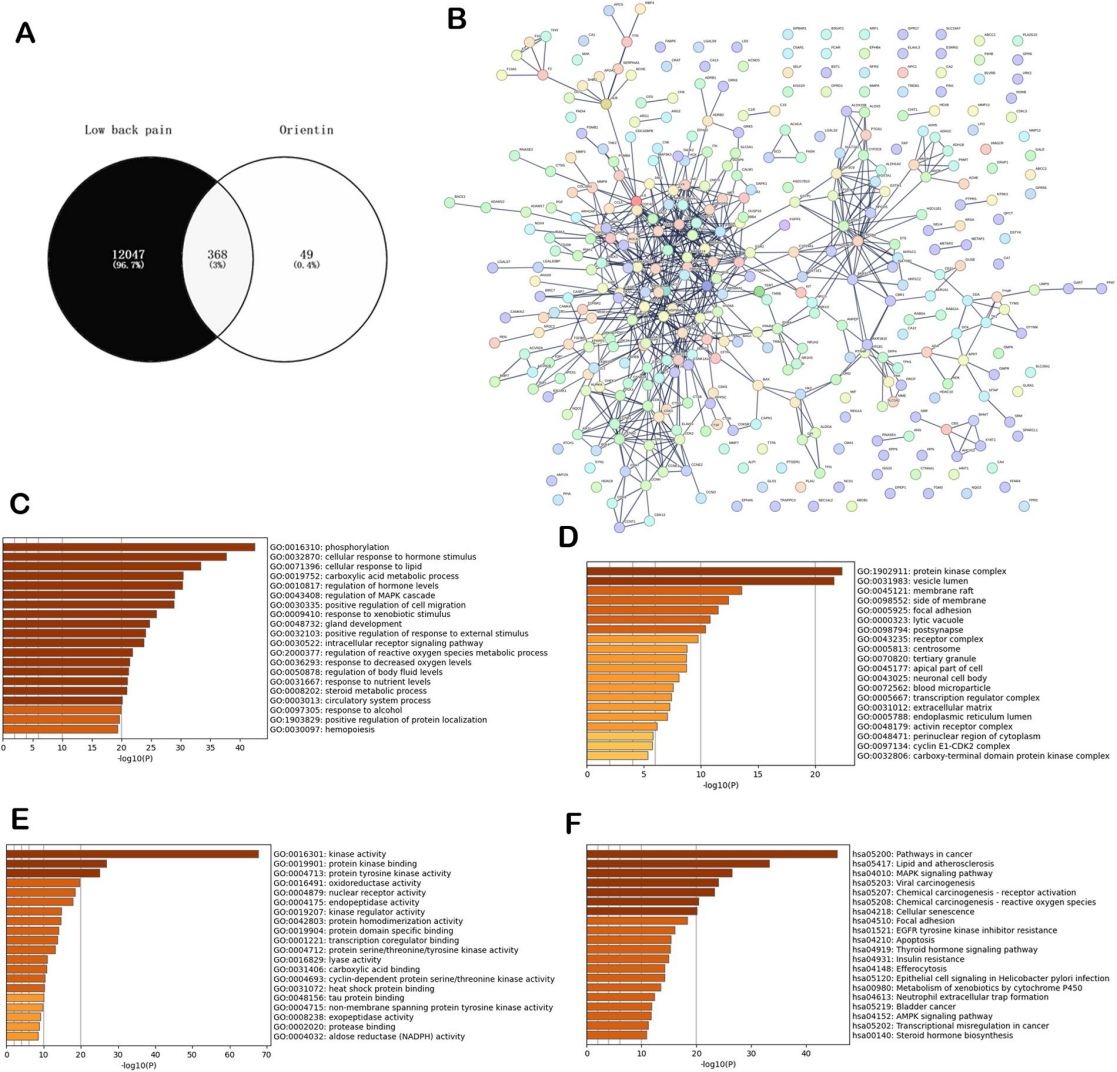
Network pharmacology analysis of orientin in low back pain. **(A)** Venn diagram of overlapping target genes between orientin and low back pain. **(B)** PPI network of the overlapping genes. **(C–E)** GO enrichment analysis ranked by −log10(P): biological process **(C)**, cellular component **(D)**, and molecular function **(E)**. **(F)** KEGG pathway enrichment analysis ranked by −log10(P).

A total of 12416 low-back pain-related targets were retrieved from the GeneCards database, and an additional 126 targets were obtained from the OMIM database. After duplicate removal, 12415 unique genes were compiled as the complete set of low-back pain-associated targets. In terms of compound, 434 orientin related targets were downloaded and 417 targets were remained after duplicate removal. There were 368 orientin-low back pain overlapped targets as shown in Venn diagram (Figure 2A).

#### 3.1.2 PPI network and candidate hub genes

As shown in Figure 2B, the PPI network contained 368 nodes and 670 interacting edges, with an average node degree of 3.67. The top 10 candidate hub genes ranked using Maximal Clique Centrality (MCC) score were SRC, EGFR, PTPN11, JAK2, PTK2, CCNA2, CDK1, IGF1R, PDGFRA, MET. In terms of degree method, the top 10 candidate hub genes were SRC, HSP90AA1, EGFR, AKT1, ESR1, MAPK1, CYP3A4, BCL2, MAPK8, CYP1A1. SRC, EGFR genes are overlapped genes in both 2 methods of MCC and degree.

#### 3.1.3 GO and KEGG pathway enrichment analysis

The top 15 items of GO-BP results mainly comprised cellular response to hormone stimulus and lipid, as shown in Figure 2C. GO-CC results mainly comprised the protein kinase complex, vesicle lumen, membrane raft, as shown in Figure 2D. GO-MF results mainly comprised the kinase activity, protein kinase blinding, protein tyrosine kinase activity as shown in Figure 2E. KEGG enrichment analysis revealed that the MAPK, TNF, AMPK and EGFR signaling pathways, as well as cytochrome P450-mediated xenobiotic metabolism, were significantly enriched (Figure 2F).

### 3.2 Candidate hub gene prediction based on machine learning

**Figure 3:**
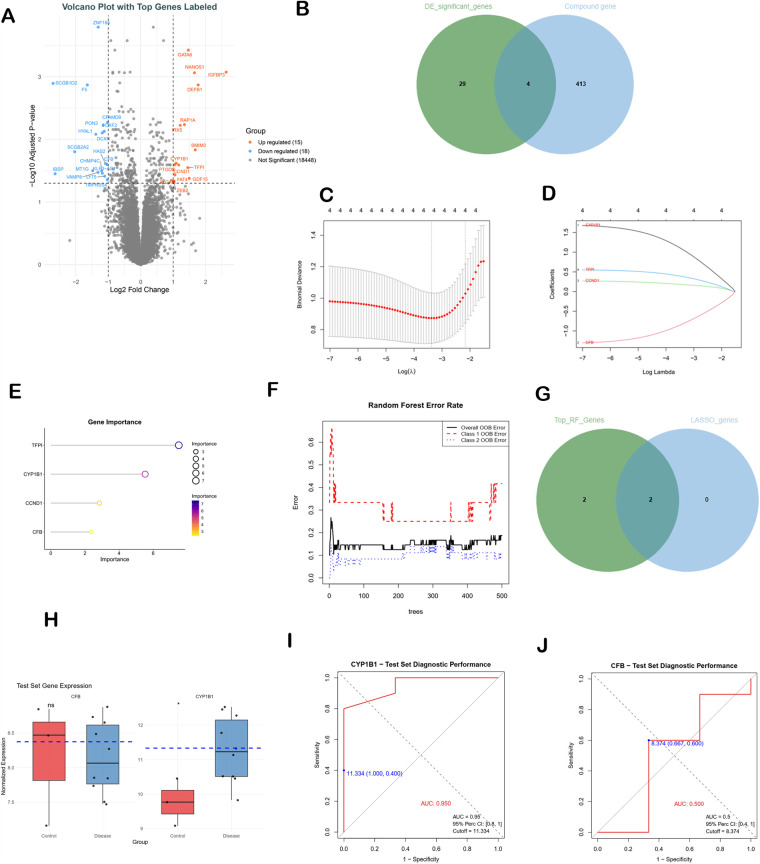
Machine learning screening of hub genes. **(A)** Differentially expressed genes in GSE70362 between low back pain and control samples. **(B)** Overlapping genes between differentially expressed genes and orientin-related targets. **(C)** Binomial deviance of LASSO regression. **(D)** Coefficient distribution of LASSO regression. **(E)** Gene importance ranking of random forest. **(F)** Error rate analysis of random forest model. **(G)** Venn diagram of intersecting genes from LASSO and random forest. **(H)** Two genes expression in test set. **(I)** AUC values of CYP1B1. **(J)** AUC values of CFB.

After normalization of the GSE70362 dataset, a total of 33 significant differentially expressed genes were identified, including 15 upregulated and 18 downregulated genes (Figure 3A). The intersection of these 33 differentially expressed genes with orientin-related target genes yielded four overlapping genes: TFP1, CYP1B1, CCND1, and CFB (Figure 3B).

Two machine learning algorithms, LASSO regression and random forest, were further applied to screen pivotal hub genes. LASSO regression filtered out two core candidate genes, CYP1B1 and CFB, according to binomial deviance and coefficient distribution (Figures 3C,D). Meanwhile, the random forest algorithm calculated gene importance and model error rate, and TFP1 and CYP1B1 showed higher importance than other candidate genes (Figures 3E,F). Venn analysis was performed to intersect the key genes obtained from LASSO regression and random forest, and two overlapping hub genes CYP1B1 and CFB were finally determined (Figure 3G).

ROC curve analysis on the independent test set showed that CYP1B1 had good diagnostic performance for IDD-associated low back pain, with an AUC of 0.950 (95% percentile CI: 0.800-1.000, *P* = 0.0179). At the optimal cutoff value of 11.334, CYP1B1 achieved a perfect specificity of 1.000 (no false positives) and a positive predictive value of 1.000, indicating that all samples predicted to be diseased were true positives. In contrast, CFB showed no diagnostic value, with an AUC of 0.500 (95% percentile CI: 0.400-1.000, *P* = 0.8930), which was equivalent to random guessing. Given its lack of diagnostic utility, CFB was excluded from further downstream analyses.

### 3.3 Immune infiltration analysis

**Figure 4:**
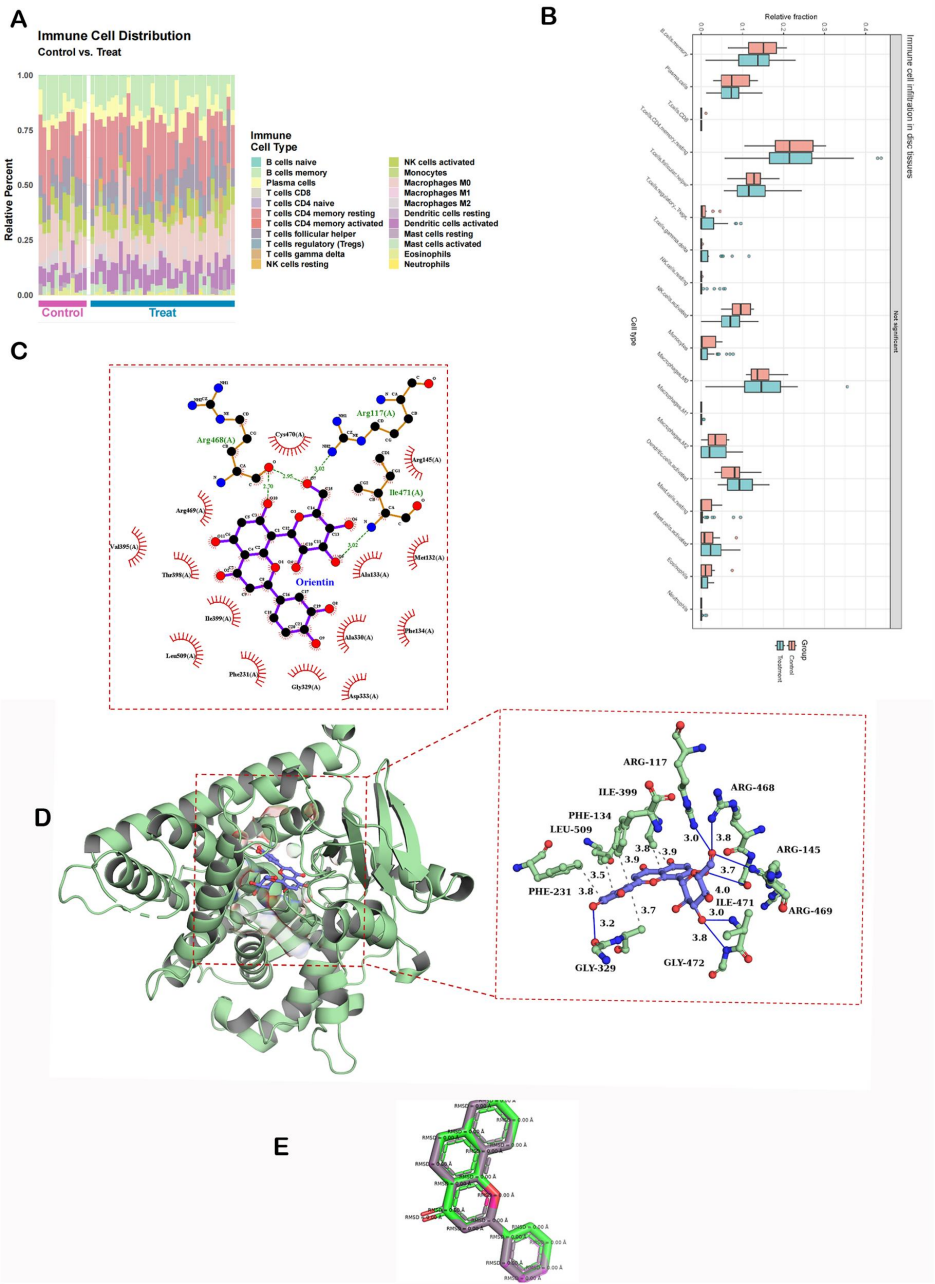
Immune infiltration analysis and molecular docking validation. **(A)** Stacked bar chart showing the relative distribution of 22 immune cell subsets in control and disease samples. **(B)** Box plots comparing the infiltration levels of individual immune cell subtypes between the two groups. **(C)** Two dimensions of docking site. **(D)** Three dimensions of docking pocket and mode: orientin (purple) and CYP1B1 (green, PDB ID: 3PM0). **(E)** Validation of the docking protocol by redocking of the native ligand *α*-naphthoflavone into CYP1B1 (PDB ID: 3PM0). The crystal structure pose of *α*-naphthoflavone is shown in green sticks, and the redocked pose obtained using AutoDock Vina (exhaustiveness = 64) is shown in magenta sticks.

As shown in Figure 4A, the stacked bar chart reflected the proportional distribution of 22 immune cell subsets in each sample. Macrophages, T cells and B cells dominated the immune landscape in both control and disease groups. Nevertheless, slight proportional differences were observed: naive B cells were more abundant in the control group, whereas CD4 memory resting T cells and M0 macrophages were slightly enriched in the disease group.

Box plots in Figure 4B illustrate the distribution of immune cell infiltration across groups. Immune cell infiltration analysis using the CIBERSORT algorithm revealed minimal baseline immune infiltration within intervertebral disc tissue, a characteristic feature consistent with the tissue's immune-privileged status. In uncorrected pairwise comparisons, only the proportion of activated NK cells showed a nominally significant difference between the IDD and control groups (raw *P* = 0.04949). However, after Benjamini-Hochberg FDR correction for multiple comparisons, no statistically significant differences in the relative proportions of any of the 22 immune cell subtypes were observed between groups (all FDR *P*-values > 0.05). This result suggests that CYP1B1 exerts its therapeutic effects directly on intervertebral disc cells rather than through modulating the immune microenvironment.

Spearman correlation analyses were performed to assess the associations between the expression of hub genes (CYP1B1 and CFB) and the infiltration fractions of immune cell subsets. In uncorrected analyses, three nominally significant correlations were identified: CYP1B1 expression was positively correlated with M2 macrophage infiltration (raw *P* = 0.0031); CFB expression was negatively correlated with resting mast cell infiltration (raw *P* = 0.0066) and positively correlated with eosinophil infiltration (raw *P* = 0.0150). However, after FDR correction for multiple testing, none of these associations remained statistically significant (all FDR *P*-values > 0.05). Therefore, these results indicated that no statistically robust correlation between CYP1B1/CFB expression and immune cell infiltration could be confirmed after accounting for multiple comparisons.

### 3.4 Molecular docking

As shown in Figure 4C, the docking energy between orientin and CYP1B1 was −9.5 kcal/mol, indicating a thermodynamically favorable interaction. Hydrophobic interactions were formed by Ala330 (3.73 Å), Ile399 (3.84/3.85 Å), and Leu509 (3.55 Å), contributing to the stabilization of the binding interface.

A complex hydrogen bond network was observed, in which Arg117 (2.29 Å), Arg145 (3.12 Å), and Arg468 (2.78/3.14 Å) formed multiple hydrogen bonds with the same oxygen atom of the ligand, suggesting cooperative binding effects. Additional hydrogen bonds were contributed by Gly329 (2.59 Å), Arg469 (3.53 Å), Ile471 (2.04 Å), and Gly472 (3.20 Å). Notably, the interaction involving Ile471 (2.04 Å) represents an ultrashort hydrogen bond, while Arg468 appears to play a key role in maintaining binding stability.

The three-dimensional structure of the docking pocket is illustrated in Figure 4D. To validate the docking protocol, the co-crystallized ligand *α*-naphthoflavone was redocked into CYP1B1, yielding a binding energy of −8.3 kcal/mol and an RMSD of 0.00 Å relative to the crystal structure, confirming the reliability of the docking procedure.

As shown in Figure 4E, the redocked pose (magenta) closely overlaps with the original crystallographic ligand (green), with an RMSD of 0.00 Å, demonstrating that the docking parameters accurately reproduce the experimentally observed binding mode.

### 3.5 Molecular dynamics simulation

**Figure 5:**
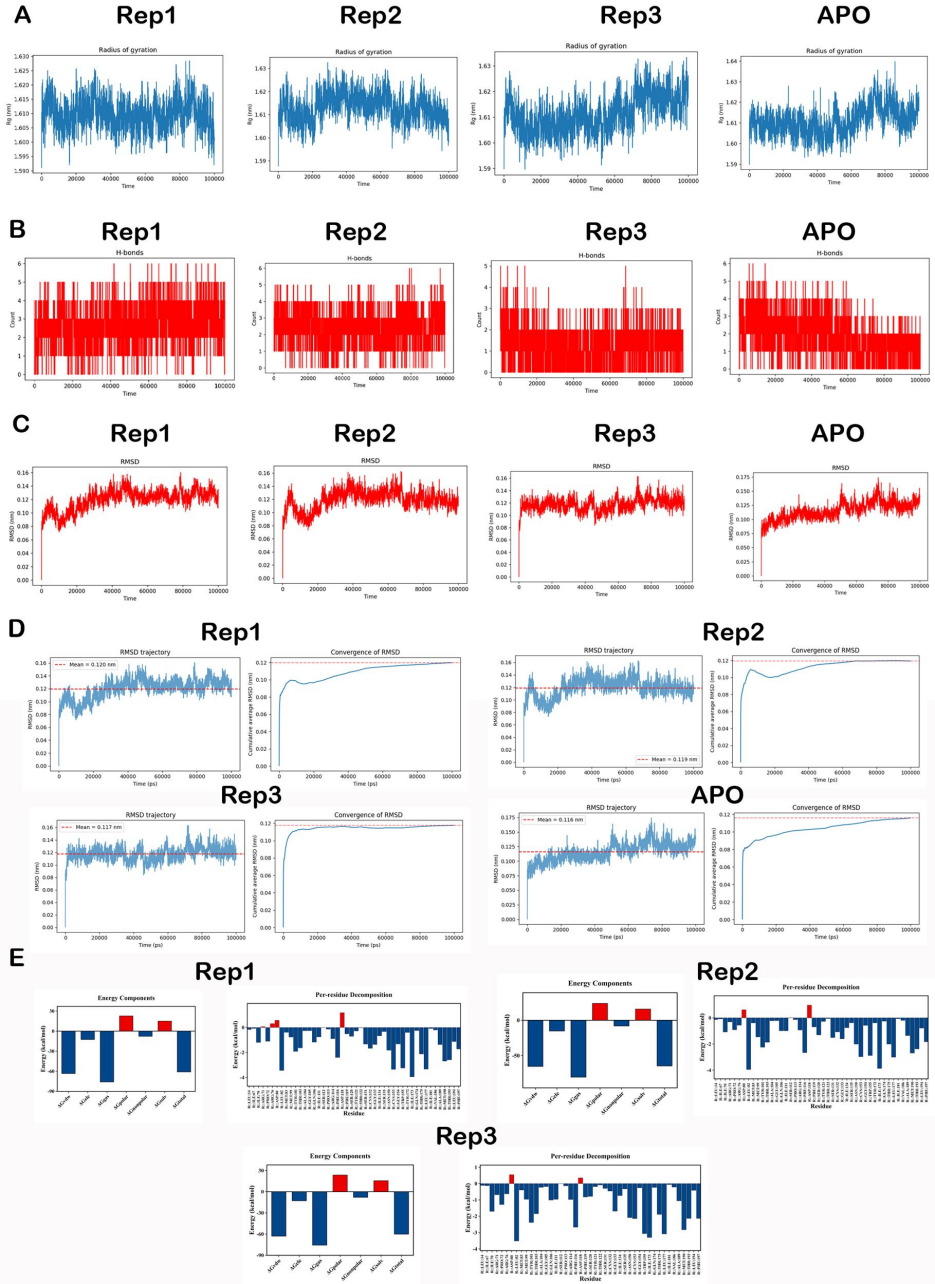
Dynamic properties of orientin-CYP1B1 complex and apo-CYP1B1 from 100 ns molecular dynamics simulations. **(A)** Radius of gyration. **(B)** Hydrogen bond number. **(C)** Backbone RMSD. **(D)** Cumulative average RMSD for convergence validation. **(E)** MM/PBSA binding free energy and residue-based energy decomposition. Quantitative analyses were performed using trajectories from 60 ns to 100 ns. Orientin binding obviously enhanced the structural stability of CYP1B1 via stable hydrogen bond interactions. Abbreviations: REP1: repeated 1, REP2: repeated 2, REP3: repeated 3, APO: apo-protein.

As shown in Figure 5, molecular dynamics simulations were performed to evaluate the structural stability of orientin-CYP1B1 complex, with three independent replicates (Rep1, Rep2, Rep3) and one apo-CYP1B1 (APO) control simulation. In Figure 5A, the radius of gyration (Rg) profiles of all systems are presented. For orientin-CYP1B1 complex (Rep1–Rep3), the Rg values remained consistently stable between approximately 1.60–1.62 nm throughout the simulation, indicating that the protein maintained a compact and well-folded structure without significant expansion or unfolding. In contrast, the apo-CYP1B1 control showed a slight but statistically significant increase in Rg over time, with a higher mean value (1.616 ± 0.006 nm vs. 1.613 ± 0.006 nm in the complex, *p* < 0.001), suggesting that the absence of orientin led to a more loosely packed protein conformation.

Figure 5B displays the hydrogen bond profiles. The orientin-CYP1B1 complex maintained a high and stable number of intermolecular hydrogen bonds, with an average of 2.243 ± 1.269 across the three replicates, and frequent formation of 3–5 hydrogen bonds throughout the simulation. These stable interactions indicate strong and persistent binding between orientin and CYP1B1. In comparison, the apo-CYP1B1 control showed fewer and less stable intramolecular hydrogen bonds, with an average of only 1.201 ± 0.858 (*p* < 0.001), reflecting weaker internal structural support in the absence of the ligand.

Figure 5C shows the backbone root mean square deviation (RMSD) trajectories. All three complex simulations exhibited rapid initial equilibration, reaching a stable RMSD plateau by approximately 40–60 ns. The mean RMSD values for the complex were 0.127 ± 0.008 nm (Rep1), 0.122 ± 0.011 nm (Rep2), and 0.122 ± 0.010 nm (Rep3), with an overall average of 0.123 ± 0.010 nm. The apo-CYP1B1 control, however, showed a gradual increase in RMSD over time, with a higher mean value of 0.128 ± 0.011 nm (*p* < 0.001), indicating greater conformational flexibility and reduced structural stability compared to the ligand-bound complex.

Figure 5D presents the convergence validation using cumulative average RMSD analysis for each simulation replicate and the apo control. The cumulative average RMSD curves for all three complex simulations plateaued after 60 ns, confirming that the systems had reached thermodynamic equilibrium. The mean RMSD values for the complex were consistent across replicates (0.116–0.120 nm), demonstrating high reproducibility. In contrast, the apo-CYP1B1 cumulative average RMSD continued to rise more slowly, reflecting a lack of full structural stabilization even at the end of the simulation.

Figure 5E shows the MM/PBSA binding free energy components and per-residue decomposition for each of the three independent orientin-CYP1B1 complex replicates. The complex exhibited favorable binding free energies across all 3 replicates, with dominant contributions from van der Waals and electrostatic interactions, while polar solvation energy contributed unfavorably. Per-residue decomposition identified several key residues in the CYP1B1 binding pocket that contributed significantly to ligand binding, consistent with the hydrogen bond and RMSD stability results. The high consistency of binding free energy values and key residue contributions across the three independent replicates further confirmed the reliability and reproducibility of the binding interaction between orientin and CYP1B1.

These results demonstrate that orientin binding significantly enhanced the structural stability of CYP1B1, as evidenced by reduced RMSD, stable Rg, and persistent hydrogen bond interactions. The three independent replicates showed consistent behavior, and the apo control confirmed that the observed stability was ligand-dependent.

### 3.6 Expression of CYP1B1 in IDD-associated low back pain

**Figure 6:**
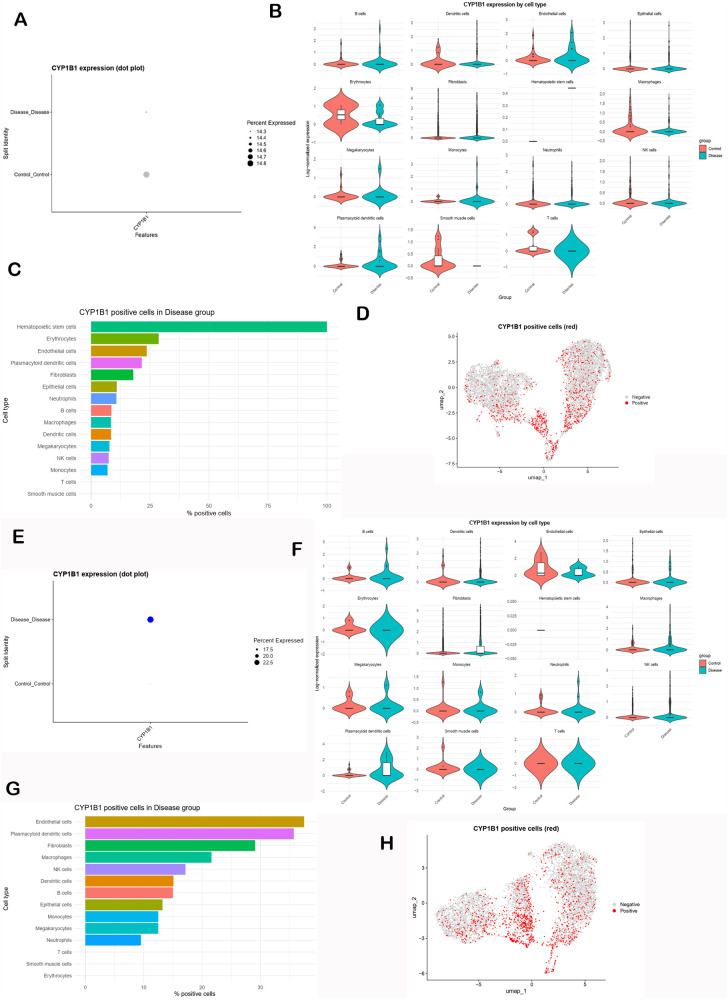
Single-cell RNA-Seq analysis of CYP1B1 expression in human intervertebral disc tissues. **(A–D)** CYP1B1 expression in nucleus pulposus (NP) tissues. **(A)** Dot plot of overall expression. **(B)** Violin plots across cell types (red: control; cyan: disease). **(C)** Percentage of CYP1B1-positive cells in disease group. **(D)** UMAP visualization of CYP1B1-positive cells (red). **(E–H)** CYP1B1 expression in annulus fibrosus (AF) tissues. **(E)** Dot plot of overall expression. **(F)** Violin plots across cell types. **(G)** Percentage of CYP1B1-positive cells in disease group. **(H)** UMAP visualization of CYP1B1-positive cells.

In human NP cells, dot plot analysis revealed that CYP1B1 expression was elevated in the disease group compared to controls (Figure 6A). Violin plots demonstrated that CYP1B1 was broadly expressed across multiple cell populations, with hematopoietic stem cells and smooth muscle cells exhibiting the most prominent upregulation in disease conditions (Figure 6B). Quantification of CYP1B1-positive cells in the disease group showed that hematopoietic stem cells harbored the highest proportion of CYP1B1-positive cells, approaching 100%, followed by erythrocytes, endothelial cells, and plasmacytoid dendritic cells (Figure 6C). UMAP visualization confirmed widespread distribution of CYP1B1-positive cells across the NP cell landscape, with enrichment in clusters consistent with the quantitative findings (Figure 6D). Wilcoxon rank-sum test with FDR correction was used to assess CYP1B1 expression differences between disease and control groups across 15 cell types. Only fibroblasts showed a statistically significant difference in CYP1B1 expression after FDR correction (*P* = 0.0029, q = 0.0401). Macrophages and NK cells exhibited nominally significant raw *p*-values but did not survive FDR correction, while the remaining 11 cell types showed no significant expression differences between groups.

Parallel analysis was performed on annulus fibrosus (AF) cells following the completion of NP cell characterization. In contrast, analysis of AF cells revealed distinct patterns of CYP1B1 expression and distribution. Dot plot analysis indicated a notable increase in CYP1B1 expression in the disease group compared to controls (Figure 6E). Violin plots showed that while CYP1B1 was detectable across various cell types, endothelial cells and plasmacytoid dendritic cells displayed the most pronounced upregulation in the disease group (Figure 6F). Quantification of CYP1B1-positive cells in the disease group demonstrated that endothelial cells and plasmacytoid dendritic cells contained the highest percentages of positive cells, followed by fibroblasts and macrophages (Figure 6G). UMAP visualization confirmed the presence of CYP1B1-positive cells across the AF cell landscape, with enrichment in specific clusters corresponding to the quantitative results (Figure 6H). In AF cells, fibroblasts were also the only significantly altered cell type, with an extremely high level of statistical significance (*P* = 2.96 × 10⁻^3^⁰, q = 3.84 × 10⁻^2^⁹). In both tissues, all other cell types showed no significant expression differences between groups after FDR correction.

These results confirm that fibroblasts are the primary cell population with disease-associated CYP1B1 expression changes in both NP and AF tissues, with a far more pronounced effect in AF cells. In contrast to the AF tissue, analysis of the NP scRNA-seq dataset revealed an overall trend of decreased CYP1B1 expression in disease samples, these data indicate that CYP1B1 expression is dynamically regulated in a cell-type-specific and tissue-specific manner under disease conditions. The preferential upregulation of CYP1B1 in distinct cell populations within NP versus AF cells suggests potential divergent functional roles of this gene in the pathogenesis of IDD.

### 3.7 Cell experiments validation

**Figure 7:**
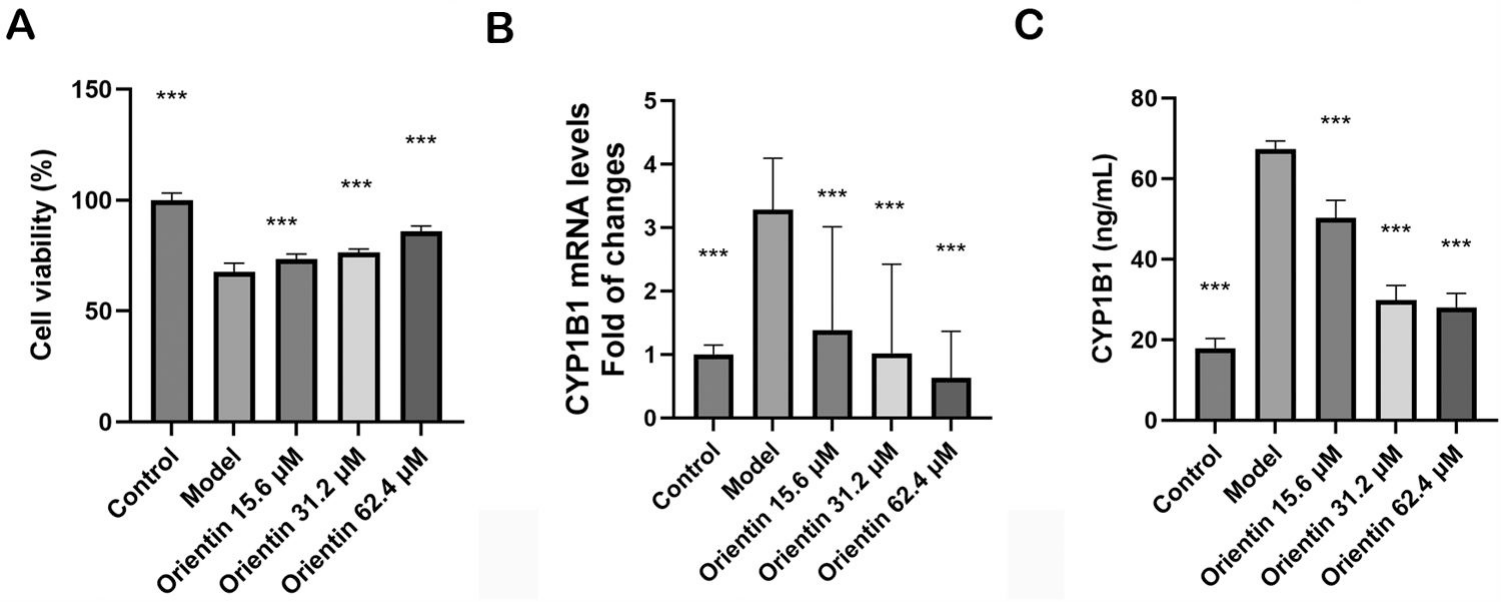
*In vitro* cell experimental validation. **(A)** Cell viability of human annulus fibrosus cells cells. **(B)** RT-PCR detection of CYP1B1 mRNA expression. **(C)** ELISA detection of CYP1B1 expression.

Cell viability was markedly reduced in the IL-1β-stimulated model group relative to untreated control cultures (Figure 7A), while graded orientin supplementation dose-dependently rescued cell survival. Consistent trends were observed for CYP1B1 transcript abundance, elevated CYP1B1 mRNA expression was detected within the IL-1β model group compared with controls, and incubation with increasing orientin concentrations suppressed CYP1B1 transcription across all three treatment cohorts (Figure 7B). At the protein level, CYP1B1 protein content was upregulated in IL-1β-stimulated cells versus control samples. Orientin treatment concentration-dependently lowered CYP1B1 protein abundance in all three dosing groups (Figure 7C).

## 4 Discussion

Low back pain is a major global health problem that predominantly affects adults and older individuals. IDD is widely recognized as a principal contributor to low back pain and is closely associated with inflammation, oxidative stress, dysregulated autophagy, fibrosis, and excessive mechanical loading (35). The pathological mechanisms underlying IDD involve an imbalance between extracellular matrix catabolism and anabolism within the intervertebral disc, altered spinal biomechanics, DNA damage, oxidative stress, and cellular senescence (36). In addition, cytokine mRNA levels in human lumbar disc biopsies have been shown to correlate positively with low back pain (37), highlighting the importance of inflammatory signaling in disease progression. Recent studies have continued to identify additional intrinsic mechanisms involved in low back pain. CYP1B1 is a dioxin-inducible oxidoreductase belonging to the cytochrome P450 superfamily and participates in the metabolism of estradiol, retinol, tamoxifen, melatonin, sterols, and other endogenous or exogenous substrates. CYP1B1 has distinctive biological characteristics: it is expressed in multiple tissues and contributes broadly to metabolic regulation. The CYP1B1 gene has been implicated in primary open-angle glaucoma, hormone-related cancers (38), and several metabolic diseases (39). Importantly, the CYP1B1-oxylipin-TRPA1 axis has been reported to promote neuropathic pain through TRPA1 channel activation (40). In addition, expression quantitative trait locus analysis combined with summary-data-based Mendelian randomization using blood samples suggested that CYP1B1 may modulate neuropathic pain after spinal cord injury (41). These findings support a potential role for CYP1B1 in pain-related conditions.

Several molecular targets and signaling pathways of orientin have previously been reported, including the MAPK signaling pathway (42), PI3 K/AKT signaling pathway (43), AMPK signaling pathway (44), and Nrf2/GPX4 signaling pathway (45). Previous work also demonstrated that orientin modulates the AMPK/SIRT1 pathway in rat nucleus pulposus cells *in vitro* and attenuates IDD *in vivo* (17). However, the targets of orientin across different diseases, particularly pain-related conditions, remain incompletely characterized. A prior GEO and immune infiltration analysis using the GSE124272 dataset suggested that CYP27A1, FAR2, and CYP1B1 may be candidate hub genes related to IDD-associated low back pain (46). In the present study, we applied a more comprehensive strategy that integrated two machine learning algorithms based on the independent GSE70362 dataset, network pharmacology, immune infiltration analysis, molecular docking, and molecular dynamics simulation. Four candidate hub genes, TFP1, CYP1B1, CCND1, and CFB, were identified, and CYP1B1 was prioritized because of its consistent performance across the two machine learning algorithms and its diagnostic AUC. In addition, KEGG prediction from the network pharmacology analysis indicated that cytochrome P450-related metabolism, which is directly linked to CYP1B1, may represent a relevant pathway through which orientin affects low back pain. Collectively, these analyses highlighted CYP1B1 as a potentially important target of orientin in IDD-associated low back pain. This prediction was further supported by molecular docking, which showed favorable binding energy between orientin and CYP1B1, and by molecular dynamics simulation, which suggested a stable orientin-CYP1B1 complex. Moreover, the *in vitro* experiments showed that orientin regulated CYP1B1 expression, providing new evidence that may expand the current understanding of the molecular mechanisms by which orientin acts in pain-related or degenerative diseases.

This study has several strengths. First, multiple complementary prediction and validation approaches were employed to identify candidate hub genes linking orientin with IDD-associated low back pain, which strengthens the reliability of the screening process compared with studies relying only on traditional network pharmacology. Beyond conventional network pharmacology, machine learning can improve discovery and decision-making from GEO datasets (47). Previous studies have shown that machine learning facilitates the identification of oxidative stress-related diagnostic markers (48) and immune-related candidate hub genes (49). In the present study, machine learning helped prioritize CYP1B1 from the four predicted candidate hub genes, and subsequent validation further supported a close relationship between orientin and CYP1B1.

Several limitations should also be acknowledged. First, all experimental validation was performed exclusively in an *in vitro* IL-1β-stimulated human annulus fibrosus cell model, which cannot fully reproduce the complex *in vivo* pathophysiology of IDD-associated low back pain, including mechanical loading, multicellular crosstalk, and nociceptive signaling. No *in vivo* animal model validation was conducted. Second, direct biochemical evidence of orientin-CYP1B1 target engagement is still lacking. Molecular docking and molecular dynamics simulations provide structural predictions, but confirmatory biophysical assays, such as surface plasmon resonance or isothermal titration calorimetry, are needed. Third, causal perturbation experiments, including CYP1B1 knockdown, overexpression, and rescue assays, were not performed; therefore, the present findings support an associative rather than causal relationship between CYP1B1 and the effects of orientin. Fourth, the relatively small sample size of the GSE70362 dataset and the absence of an independent external validation cohort may limit the generalizability of the machine learning results. The independent test set was small (*n* = 13), and the high AUC of CYP1B1 should therefore be regarded as exploratory; the selected cutoff also prioritized specificity at the cost of sensitivity. Fifth, the single-cell RNA-seq comparisons were limited to one disease sample and one control sample for both nucleus pulposus and annulus fibrosus tissues, which cannot account for inter-individual variability. Although the large number of cells per sample produced extremely small *p*-values for differentially expressed genes, such as *P* = 2.96 × 10⁻^30^, this single-sample design means that the observed expression patterns should be considered preliminary. Larger single-cell cohorts with multiple biological replicates are required to confirm these findings. Taken together, these limitations indicate that CYP1B1 should currently be considered a candidate target of orientin that warrants further rigorous functional validation.

## 5 Conclusion

In summary, this study integrated the machine learning, network pharmacology analysis, immune infiltration and founded that the CYP1B1 is a potential candidate hub gene of orientin to IDD-associated low back pain. Further the combination of orientin and CYP1B1 was validation of molecular docking, molecular dynamics simulation and cell experiments. This study revealed that CYP1B1 is a potential candidate hub gene for low back pain and orientin may regulate the IDD-associated low back pain via CYP1B1 gene.

## Data Availability

The original contributions presented in the study are included in the article/Supplementary Material, further inquiries can be directed to the corresponding author.
